# Prediction of Unmet Primary Care Needs for the Medically Vulnerable Post-Disaster: An Interrupted Time-Series Analysis of Health System Responses 

**DOI:** 10.3390/ijerph9103384

**Published:** 2012-09-25

**Authors:** Jennifer D. Runkle, Hongmei Zhang, Wilfried Karmaus, Amy Brock-Martin, Erik R. Svendsen

**Affiliations:** 1 Nell Hodgson School of Nursing, Emory University, Atlanta, GA 30322, USA; 2 Department of Epidemiology & Biostatistics, Arnold School of Public Health, University of South Carolina, Columbia, SC 29208, USA; Email: hzhang@mailbox.sc.edu (H.Z.); karmaus@mailbox.sc.edu (W.K.); esvendse@tulane.edu (E.R.S.); 3 Health Services, Policy, and Management, University of South Carolina, Columbia, SC 29208, USA; Email: brocka@mailbox.sc.edu; 4 South Carolina Rural Health Research Center, Columbia, SC 29210, USA; 5 Department of Environmental Health Sciences, Tulane University School of Public Health and Tropical Medicine, New Orleans, LA 70112, USA

**Keywords:** technological disaster, vulnerable populations, access to care, ambulatory care sensitive conditions, secondary surge capacity, recovery, health system, forecast modeling

## Abstract

Disasters serve as shocks and precipitate unanticipated disturbances to the health care system. Public health surveillance is generally focused on monitoring latent health and environmental exposure effects, rather than health system performance in response to these local shocks. The following intervention study sought to determine the long-term effects of the 2005 chlorine spill in Graniteville, South Carolina on primary care access for vulnerable populations. We used an interrupted time-series approach to model monthly visits for Ambulatory Care Sensitive Conditions, an indicator of unmet primary care need, to quantify the impact of the disaster on unmet primary care need in Medicaid beneficiaries. The results showed Medicaid beneficiaries in the directly impacted service area experienced improved access to primary care in the 24 months post-disaster. We provide evidence that a health system serving the medically underserved can prove resilient and display improved adaptive capacity under adverse circumstances (*i.e.*, technological disasters) to ensure access to primary care for vulnerable sub-groups. The results suggests a new application for ambulatory care sensitive conditions as a population-based metric to advance anecdotal evidence of secondary surge and evaluate pre- and post-health system surge capacity following a disaster.

## 1. Introduction

At the complex interface between human factors (e.g., design flaw, negligence) and technology lies the unremitting possibility of a technological disaster. Technological disasters involve a breakdown in manmade systems (e.g., airplane crash, chemical spill, failure of a dam, and leakage of industrial contaminants) that often occur without warning, and may go unnoticed for some time by those affected [1,2,3]. Large-scale public health emergencies have the potential to overwhelm the local health care system at multiple time points including the acute response period and throughout recovery and reconstruction. In the midst of persistent threats in bio-terrorism, natural and technological disasters, and other large-scale public health emergencies, questions arise concerning “everyday preparedness” and the state of surge capacity for health systems across the nation. Surge capacity is defined as the ability of a health system to meet the sudden or extended increase in demand for medical care in the event of a crisis [4,5,6]. Disaster planners should anticipate fluctuation in surge patterns throughout disaster response and recovery efforts, not merely a spike in volume during the acute response. We argue that dependent upon magnitude and the baseline health status of the affected population, disasters may precipitate sustained secondary surge patterns. Secondary surges are signified by amplification in demand for medical services in the weeks, months, or years following the event. We describe secondary surge capacity as the ability of a health care system to respond to the surging and fluctuating volume in medical care needs throughout the extended response period. 

### 1.1. Secondary Surge Capacity Metric

The Institute of Medicine (IOM) has called for metrics to measure the effectiveness of public health response and recovery efforts to guide decision-making that ensures recovery for vulnerable populations [7]. Standardized metrics are necessary to advance anecdotal evidence of the secondary surge phenomenon into objective data. Population-based metrics lay the groundwork for public health monitoring of the effects of surge on health system response and thereby, patient-care capacity, quality, access, and health outcomes [8]. A growing body of research has documented secondary surges in primary care volume in the weeks and months post-disaster, especially for chronic medical conditions and non-acute ambulatory health needs, suggesting that provision of primary care services is a critical, but impromptu component in disaster recovery [9,10,11,12,13,14,15,16,17,18,19]. Insufficient access to primary care services during the secondary surge likely drives health disparities in vulnerable subgroups (e.g., unemployed, un(der)insured, Medicaid beneficiaries, racial/ethnic minority groups, unemployed, low-income and medically underserved populations), complicating post-disaster recovery efforts [19,20,21,22]. Metrics quantifying the secondary surge impact on health care access for vulnerable populations post-event are undefined in the disaster literature [8]. 

### 1.2. Natural Experiment

We propose the use of an unusual natural experiment involving a chlorine spill in Graniteville, South Carolina (SC, USA) to quantify the long-term effects of a technological disaster on access to primary care for a medically underserved population. The small unincorporated town of Graniteville made national headlines on 6 January 2005 when a train derailed and discharged an estimated 60 tons of chlorine into the unsuspecting community. Approximately 5,400 residents within a one-mile radius of the spill were subject to a mandatory evacuation the following day and were not permitted home until 1–2 weeks later. As a result of injuries incurred from direct chlorine inhalation, nine individuals died and at least 550 residents sought immediate medical assistance. To date, the community is still combating the ongoing physical, psychological, and economic traumas surrounding the devastation. The town has been designated by the Health Services Research Administration as a medically underserved area/population (MUA/P) for having insufficient number of primary care providers, elevated infant mortality, with a disproportionate number of the population that is living in poverty and/or elderly [23]. A technological disaster’s impact on access to primary care for vulnerable sub-groups within a MUA community is unknown; whereby secondary surge patterns in primary care need is not well characterized in the disaster literature [22]. 

### 1.3. Study Objectives

Public health surveillance in the months following a technological disaster has generally focused on monitoring latent health and environmental effects, not a health systems’ capacity to provide necessary medical services for at-risk populations [24,25,26]. This study explored the practical application of continued medical surveillance of Ambulatory Care Sensitive Conditions (ACSCs), a widely cited indicator of primary care access. We propose the use of preventable ACSCs to estimate unmet primary care need in Medicaid beneficiaries directly impacted and recovering from a technological disaster. We sought to generate forecasts for the number of ACSC visits after the 2005 chlorine spill in order to estimate expected number of primary care visits that might be anticipated in future technological disasters affecting MUA/Ps. The research will address a gap in understanding a disaster’s long-term impact on changes in demand for primary care services and subsequent entry of vulnerable disaster populations into a health care system responding to changes in demand. 

## 2. Methodology

Data used in the study was monthly ACSC counts for hospital and ED visits combined. Hospital and ED discharge data for SC Medicaid beneficiaries age 18 to 64 were obtained from the SC Medicaid paid UB-92 claims housed by the Office of Research and Statistics (ORS) of South Carolina State Budget and Control Board. A randomly generated de-identified linker based on the Medicaid recipient number was assigned to each encounter, permitting a count of individual patients and ED/hospital discharges. ORS estimated that more than 99.5% of Medicaid hospitalization and ED records were successfully linked. Approval for this study was obtained from the ORS and the University of South Carolina Institutional Review Board. 

### 2.1. ACSC Metric for Unmet Primary Care Need

Primary diagnosis (ICD-9 codes) for ACSC-related [27,28] hospital and Emergency department (ED) encounters were obtained from January 2002 to December 2006 for each study group. The period of January 2002 to December 2004 was characterized as the pre-disaster period and January 2005 to December 2006 as the post-disaster period. ACSC-related hospital and ED visits were combined into a summary measure of total ACSC specific counts, the primary outcome variable of interest. ACSCs are a well-known group of diagnoses used as a surrogate to measure access to primary care in health policy research. The assumption is that timely and effective primary care has the potential to reduce the risk of preventable hospitalizations and ED ACSC visits. Elevated ACSC rates across geographic areas or population subgroups may indicate inequities in access to health care; decreased availability of primary care services have been linked to higher admission rates for ACSCs [29,30,31,32]. We explored the use of pre and post-disaster changes in ACSCs as a population-based indicator to signal unmet primary care need for vulnerable Medicaid enrollees. Excess visits for ACSCs in the months following a disaster may indicate that certain members of the population are not receiving adequate and timely access to primary care. 

### 2.2. Setting

We included a direct and control group in our analysis. The direct group comprised beneficiaries residing in the town of Graniteville alongside two nearby communities in Aiken County, SC, directly impacted by the 2005 chlorine spill. Residents in the control group were in the same county, but further south from communities directly affected by the disaster; environmental data revealed that the chlorine gas traveled north. Both groups shared the same county-level health system capacity (e.g., number of hospital beds, federally qualified health center, same provider to population ratio, MUA and Health professional shortage area designations). 

### 2.3. Study Design and Methods

An interrupted time series analysis [33,34] approach was applied to construct intervention models forecasting unmet primary care (*i.e.*, ACSC volume) to analyze the sustained impact of the 2005 chlorine spill on the local health system. The time series for the direct group included a total of 60 monthly observations, with 36 months of pre-chlorine spill data (train derailment occurred on 5 January 2005) and a post-disaster period of 24 months. A separate 36 month pre- and 12 month post-disaster study was conducted to estimate temporal trends in primary care access for the control group. 

We followed the modeling strategy put forth by Box *et al.* [34] outlining an ARIMA (*p,d,q*) four step process: (1) identification, (2) estimation, (3) diagnosis, and (4) intervention hypothesis testing. In an ARIMA (*p,d,q*) model, the value of p corresponds to the memory of the process for preceding observations (*i.e.*, autoregressive component), d represents the number of times the series must be differenced to be stationary, while the moving average component is delineated by the value *q* and captures the memory of the process for preceding random shocks [33,34]. Models were diagnosed based on correlation analysis of residuals using Chi-square statistics χ^2^. We also used the AIC (Akaike’s Information Criterion) and SBC (Schwarz’s Bayesian Criterion) tests to compare nested models. We tested models for stationarity (*i.e.*, displays no secular trend) by using the Augmented Dickey-Fuller (ADF) Unit Root tests [33,35].

To evaluate change in access to primary care following the Graniteville train derailment, we included an intervention variable designed to capture change in the unconditional mean (detrended) ACSC rate. We chose to focus on the entire post-2005 chlorine spill time period assuming that the health system would take time to adjust to new conditions and changes in the health care environment. The disaster was expected to exact an abrupt, but continual effect on health system performance. Therefore, all post-disaster months were coded as 1 and the model chosen is an abrupt, permanent step function [36]. The following mixed autoregressive, moving average (ARMA) model included the effect of the chlorine spill (*i.e.*, I = intervention) on the dependent variable (*i.e.*, monthly ACSC visits) after January 2005: 


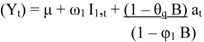
(1)

where Y_t_ is the dependent variable representing the logarithmic transformation of monthly ACSC counts (*i.e.*, monthly ACSC visits for each study group), t indexes time, μ is the mean term, ω_1 _I_1_ is the continual effect of the intervention on the dependent variable during the subsequent 24 quarters, *i.e.*, the percent change after the intervention. The right-hand side of the Equation (1) characterizes the ARIMA noise process; whereby a_t_ is the random error term. *B* is the backshift operator, θ_q_ is the coefficient of the noise model moving average factor, and φ_1_ is the coefficient of the noise model autoregressive factor.

## 3. Results

Table 1 shows the descriptive statistics for pre- and post-mean ACSC rates by study group. Mean ACSC rate in pre-disaster months was 10.8 visits per month compared to a mean ACSC post-disaster rate of 7.0 visits per month. Similarly, the pre-disaster mean rate for monthly ACSC visits was 30.0 in the control group compared to 28.0 visits per month in the post-2005 period. 

### 3.1. Intervention Model for Health Care Use Post-Chlorine Spill

Using the 36 months pre-intervention series to identify the final model, we concluded that the mixed ARMA (1,1) model was adequate for estimating the change in monthly ACSC visits of Medicaid patients in the direct group. Results from estimating Equation (1) for the direct group are shown in Table 2. The significant autoregressive term showed that shocks to the ACSC rate in the current month are felt in subsequent months. Our model showed a statistically significant moving average term indicating that an immediate past surprise to the ACSC rate has an effect on the current monthly ACSC rate. Results revealed a statistically significant negative intervention effect (regression coefficient: −0.4589; *p* < 0.001) signifying that contrary to our hypothesis the disaster produced an immediate impact and permanent reduction (37%) in ACSCs in the post-chlorine spill period. 

**Table 1:** Descriptive Statistics for Untransformed Time Series Data by study group, SC Medicaid ACSC discharges^±^ 2002–2006.

Study Group	Pre-Disaster		Post-Disaster	
	*N (months)*	Mean * (SD)	*N (months)*	Mean* (SD)
Direct	36	10.8 (4.4)	24	7.0 (3.2)
Indirect	36	30.0 (6.8)	12	28.0 (6.1)

Data source: South Carolina Office of Research and Statistics. * Mean = Monthly Mean Visits for Ambulatory Care Sensitive Conditions. ^±^ All ACSC discharge diagnosis combined for: Grand mal seizures, convulsions, severe ear, nose, and throat infections, bacterial pneumonia, cellulitis, skin grafts, gastroenteritis, kidney and urinary infection, dehydration, dental conditions, pelvic inflammatory disease, and hypoglycemia, tuberculosis, asthma, angina, diabetes, nutritional deficiencies, chronic obstructive pulmonary disease, congestive heart failure, and hypertension.

**Table 2:** ARMA (1,1) model parameter characteristics for Direct Group: SC Medicaid Beneficiaries, 2002–2006.

Parameter	Estimate	Std Error	T	*P*-value
MU	2.3108	0.0780	29.48	<0.0001
MA 1,1	−0.9642	0.1216	−7.93	<0.0001
AR 1,1	−0.8938	0.1641	−5.45	<0.0001
2005 Chlorine spill ^a^	−0.4589	0.1239	−3.70	<0.001

MU = mean term. MA1,1 = moving average of order one term. AR 1,1 = autoregressive of order one term. ^a^ intervention term.

### 3.2. Forecast Modeling of Unmet Primary Care Need

**Figure 1:**
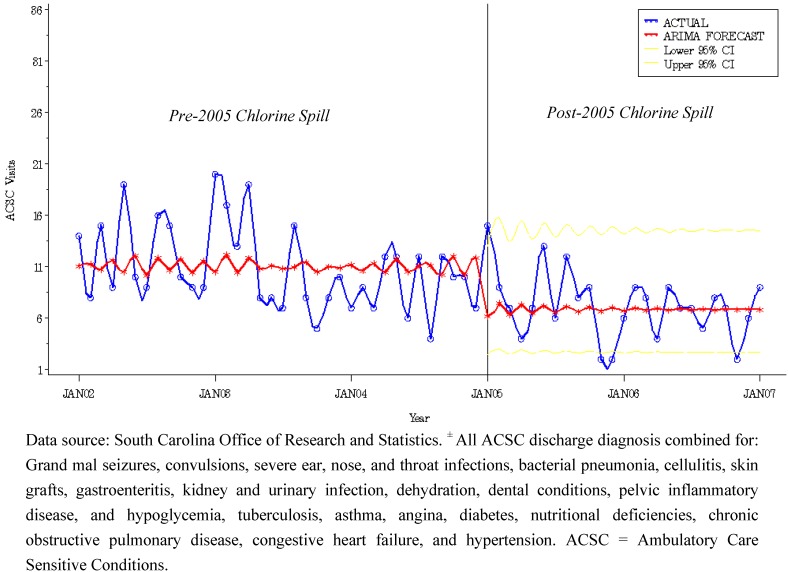
Model Actual *vs.* Predicted Plot of Ambulatory Care Sensitive Conditions ^±^ with 95% Confidence Band for Direct Group: SC Medicaid Beneficiaries, 2002–2006.

Using the ARMA model (1,1), we also forecasted future ACSC visits for Medicaid beneficiaries directly affected by the disaster. The results are shown in Figure 1. The reference line perpendicular to the horizontal axis on the plots indicates the month we started to forecast (*i.e.*, January 2005). On the left side of the line, the results are one-step-ahead predictions (*i.e.*, prediction of the *(n+1)th* value of the variable based on *n* available observations). On the right side of the line, the predictions are h-step predictions. We examined monthly visits for ACSCs 24 months ahead. 

Note that the number of ACSCs predicted by the ARIMA model after the disaster is much lower than the actual number of ACSC visits observed. At first glance, spikes in observed monthly ACSC visits above the predicted values for the post-disaster time period (*i.e.*, January 2005, June 2005, August 2005, February to March 2006, May 2006,) though not statistically significant may be perceived as a higher than expected volume for ACSC among Medicaid enrollees suggestive of unmet primary care need. However, because the model adjusts the data for momentum (autoregressive components) and persistence of random shocks (moving average components) the number of visits predicted by the model post-disaster is not exactly fit to observed values. Therefore, we can assume that the ARIMA model more accurately reflects the impact of the disaster.

### 3.3. Intervention Model for Control Community

**Figure 2:**
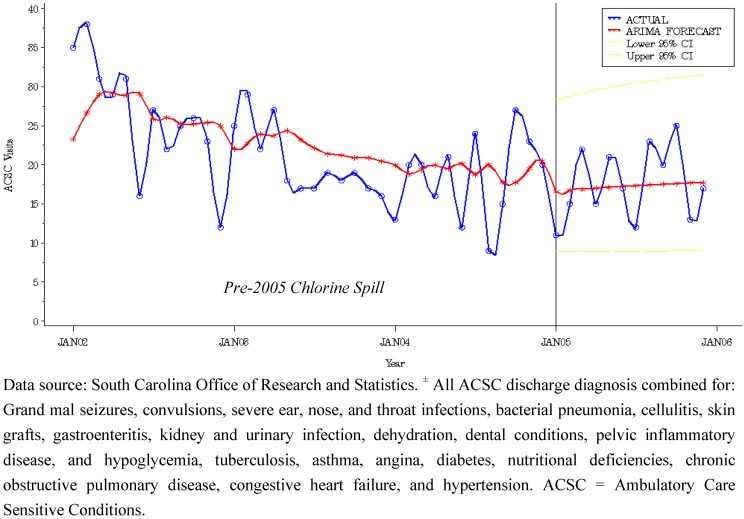
Model Actual *vs.* Predicted Plot of Ambulatory Care Sensitive Conditions ^±^ with 95% Confidence Band for Control and Control Group: SC Medicaid Beneficiaries, 2002–2006.

For evaluation and comparison purposes, we fit monthly ACSC data for the control group using the same ARMA (1,1) model in Equation (1). The estimated coefficient for the 2005 chlorine spill intervention variable was found not to be statistically significant for control communities (Table 3, Figure 2). Results from the comparison group model can be used to rule out secular changes in ACSC volume and provide support that we arrived at an appropriate inference concerning the 2005 train derailment (intervention) impact on the health system in the direct group. 

**Table 3:** ARMA (1,1) model parameter characteristics for Control Group: SC Medicaid Beneficiaries, 2002–2006.

Control Group	Estimate	Std Error	T	*P*-value
MU	3.0945	0.1434	21.58	<0.0001
MA 1,1	0.7565	0.2066	3.66	<0.001
AR 1,1	0.9285	0.1109	8.38	<0.0001
2005 Chlorine spill ^a^	−0.2228	0.2083	−1.07	0.29

MU = mean term. MA1,1 = moving average of order one term. AR 1,1 = autoregressive of order one term. ^a^ intervention term.

To provide an interpretable measure of effect size for the statistically significant intervention effect, we calculated the “proportion of systematic variance explained by the model (*R^2^*) as one minus the sum of square residuals divided by the sum of squared Y_t_ values, where Y_t_ is the difference-adjusted dependent variable” [22], which displays variance explained by the model as a proportion of total variance in ACSC rate. Thus, 21% of the variance in the intervention analysis is explained by the ARMA (1,1) model with an abrupt, permanent effect. 

### 3.4. Primary Care Visits to FQHC

**Figure 3:**
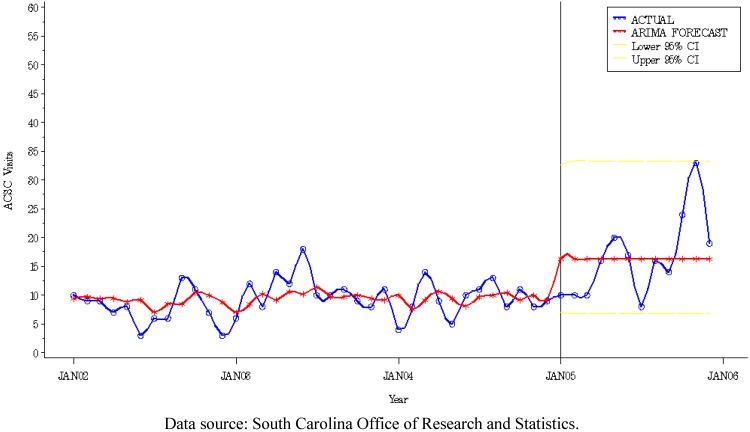
Model Actual *vs.* Predicted Plot of Primary Care Visits to a Federally Qualified Health Center with 95% Confidence Band for Direct Group: SC Medicaid Beneficiaries, 2002–2006.

A supplemental intervention analysis was performed on primary care visits for Medicaid beneficiaries in the direct group. We examined local primary care attendance at a clinic, Federally Qualified Health Center (FQHC), Rural Health Center (RHC), and physician’s office. Findings revealed a significant intervention term (e^intervention term (0.5493)) for a post-disaster increase (73%) in primary care visits to aFQHC (Figure 3). Increased patient volume redirected to a local FQHC following the chlorine spill may have served to absorb excess health system strain, while providing an invaluable safety net to Medicaid Beneficiaries seeking primary care during the secondary surge. 

## 4. Discussion

The results of this study showed that Medicaid beneficiaries in the direct group experienced improved access to timely and effective primary care in the 24 months post-disaster. This study assessed the long-term impact of an environmental disaster on primary healthcare utilization for a health system serving the medically underserved. Our results are an original contribution to the disaster epidemiology literature. We provide evidence that a health system serving a MUA can prove resilient and display improved adaptive capacity under adverse circumstances (*i.e.*, technological disaster) to ensure access to primary care for vulnerable sub-groups. Controlling for recent surprises in the local health system, we conclude that the permanent reduction in mean ACSC visits post-disaster was likely due to the impact of recovery activity associated with the chlorine spill and is not likely due to an alternative unobserved factor. 

We believe reliance on the local FQHC post-event buffered the disaster’s impact on health system performance by providing an alternate site of preventive and primary care for low-income residents directly affected by the chlorine spill. A disaster declaration was denied following the accident and therefore no federal funds were issued to aide response efforts. The local health department and response personnel were focused on the initial characterization of the event for the first two months following the spill. In March 2005, three town-hall meeting were convened with an expert panel to answer the questions of community members. At that time, local officials provided outreach information on general resources within the community, including mention of a FQHC as a medical resource. Note the immediate increase in FQHC attendance for April and May 2005 (Figure 3). In June a health registry [37] was initiated and health screenings were made available to registrants within 1 mile [38] in late August for 10 consecutive weeks. Each screened community member was provided a local community resource flier that included information on the area FQHC and if necessary, screened registrants were referred for follow-up medical or mental health care. Note the subsequent spike in FQHC visits in November 2005 following the conclusion of health screenings and patient referrals (Figure 3). Our results corroborate previous research that medically underserved populations (MUP) served by a FQHC have significantly lower ACSC rates compared to MUA populations without an FQHC [39]. 

### 4.1. Strength and Limitations

One strength of our study design is that autoregressive integrated moving average (ARIMA) models are a robust method for assessing ecological changes at the population level. We included a nonequivalent control group to account for secular changes in ACSC visits over time and to rule out threats to internal validity (e.g., history, maturation, testing, instrumentation, and regression) [33]. In the case of a natural experiment (e.g., a disaster), randomization of cases and controls is often not possible. Therefore we improved upon the non-random control group design by including successive monthly observations before and after the intervention (*i.e.*, serial pre-test and post-test measures). One additional advantage to using Medicaid data was the ability to track health care visits within and outside the enrollee’s service area, including visits in surrounding counties and states. Therefore, we were able to capture all ACSC-related discharges and primary care visits independent of service location.

There are a few limitations to our study. Due to small sample size, monthly ACSC rates could not be analyzed separately for ACSC-related hospital or Emergency department (ED) discharges. Combining ACSC visits possibly underestimated the individual impact of the disaster on ACSC volume for hospitals and EDs separately. However, the routine collection of ACSC admissions in hospital administrative data may be used as a public health surveillance measure to estimate population changes in unmet need throughout disaster recovery. Surveillance data can also be compared to ACSC benchmarking in other areas or hospitals, as ACSCs are reported at the local, county, state, and federal levels. Our results also may not be generalizable to other MUA/Ps without a local FQHC. Finally, additional factors affecting monthly ACSC rates should be accounted for in future models, including primary care provider shortages, individual characteristics, disease burden, and the estimation of unmet demand absorbed in receiving areas (neighboring counties, states, and distant areas). 

### 4.2. Directions for Future Research

This study is a preliminary step in understanding a disaster’s impact on the performance capacity of the impacted health system(s). More research is needed to test the application of ACSC as a metric for primary care access in other vulnerable disaster populations, including the uninsured and privately insured. Health system characteristics (e.g., hospital bed availability, primary care provider supply, MUA/HPSA, and availability of a FQHC or Rural Health Center (RHC)) and socio-ecologic characteristics of the population (e.g., household income, poverty, and unemployment) should be included in intervention studies as explanatory variables to help capture the context of health service utilization for future health response planning. Retrospective analysis should be performed in areas experiencing repeat occurrences of specific seasonal disaster events including flooding, tornadoes, and hurricanes to establish the ACSC as a reliable marker of access and explore the impact of persistent health system strain on adaptive capacity across disaster populations and scenarios. 

The safety net system, a system designed to ensure health care access for the most vulnerable, is not currently addressed in recovery plans. For the coordination and provision of equitable long-term primary care, we propose that areas designated as medically underserved and/or with health professional shortages (e.g., MUAs, HPSAs) incorporate a safety net response plan to ensure operational continuity throughout recovery efforts. This plan would include the expansion of local safety net services (*i.e.*, community health centers, FQHCs, rural health centers) for up to one year post-disaster. Input from local safety net providers, hospitals, and health systems alongside the residents they serve are needed during the planning process to ensure long-term provision of medical care for vulnerable populations, provider support, response capabilities, and post-event implementation. Commitment to, routine exercise of, and regular updates to the safety net plan would allow communities to prospectively better assess baseline health disparities, risk level, community priorities, and medical resources. Ultimately, without a safety net plan communities can do little more than react in the event of a public health catastrophe, resulting in poor decision making, encumbered post-disaster action, and the compromised health of vulnerable residents.

## 5. Conclusions

Public health practice has not yet addressed a gap in forecasting models that predict primary care needs for vulnerable populations in recovery from a technological disaster. Vulnerable subgroups have sustained needs in a health system that may be operating at maximum surge capacity. Slight perturbations in availability of medical services and system overload can have direct health consequences for these populations during the secondary surge. Anticipating increased primary care visits could better prepare health systems to effectively manage patient load and improve access to primary care services for all members of the affected population. Hospital administrative data on ambulatory care sensitive conditions may be used as a reproducible benchmark to quantify and predict secondary surge effects on health system access throughout disaster recovery. Results of this study illustrate the need for more research on long-term access to primary care metrics (e.g., ACSCs) for vulnerable subpopulations in recovery. Preventable visits for ACSCs may advance public health practice and surveillance efforts throughout recovery efforts by identifying healthcare disparities within the affected population. As the amount of research findings grow in this area, attention should shift to developing and disseminating evidence-based response planning measures that include prolonged surveillance and expansion of primary care services at least one year post-disaster.
